# Effects of Closed Kinetic Chain Exercises in Chemotherapy-Induced Peripheral Neuropathy: A Case Report

**DOI:** 10.7759/cureus.62732

**Published:** 2024-06-19

**Authors:** Nikita H Seth, Raghumahanti Raghuveer, Moh'd Irshad Qureshi

**Affiliations:** 1 Neurophysiotherapy, Ravi Nair Physiotherapy College, Datta Meghe Institute of Higher Education and Research, Wardha, IND

**Keywords:** quality of life, gynecological cancers, closed kinetic chain exercises, physiotherapy, chemotherapy induced peripheral neuropathy

## Abstract

Chemotherapy-induced peripheral neuropathy (CIPN) is a common adverse reaction to many first- and second-line chemotherapy medications that can be debilitating, severe, and often dose-limiting. Treatment options for CIPN are limited. We report a case of a 52-year-old female patient with Stage II ovarian cancer who was hospitalised in the chemotherapy ward for a second round of chemotherapy. We describe the effectiveness of closed kinetic chain (CKC) exercises for the management of CIPN symptoms. The patient was advised to take neurophysiotherapy. The patient complained of pain, tingling in both feet, weakness in the lower limbs, and trouble keeping her balance while walking. Thus, three days after the start of the chemotherapy drugs, physical therapy rehabilitation was started. The patient stated total pain reduction and a noticeable improvement in tingling and numbness in both lower extremities following four weeks of physical therapy. Even though CIPN usually disappears gradually over time, it can persist for an extended period. It seems doubtful that this was a spontaneous resolve, given the regularity of her symptoms before starting physiotherapy sessions and their quick recovery with treatment. Further investigation is required to comprehend the role that physiotherapy and non-pharmacologic interventions play in ameliorating CIPN symptoms and to ascertain if improvements in CIPN symptoms are associated with an increase in blood flow directly or indirectly.

## Introduction

Cancer patients receiving chemotherapy frequently suffer from the disabling side effect known as chemotherapy-induced peripheral neuropathy (CIPN). It appears as peripheral nerve injury, which results in dysfunction of the motor, sensory, and autonomic nervous systems [1]. Chemotherapy medications are vital to fight against cancer, but they can have a detrimental influence on a patient's quality of life and treatment results because of their negative effects on the neurological system [2]. The length of treatment and the chemotherapy protocol employed affect the frequency of CIPN. Up to 68% of cancer patients receiving chemotherapy may experience it, and some medications put them at higher risk than others [3]. Medications based on platinum (cisplatin, oxaliplatin), taxanes (paclitaxel, docetaxel), vinca alkaloids (vincristine, for example), and proteasome inhibitors (bortezomib, for example) are frequently responsible for CIPN [4].

Chemotherapy medications can harm healthy cells, particularly peripheral nerves, in addition to their cytotoxic effects on cancer cells that divide quickly. Drugs based on platinum have been reported to induce sensory neuropathy, which can cause pain, tingling, and numbness in the hands and feet. The main side effects of taxanes include sensory and motor neuropathy, which can cause neuropathic pain, paralysis, and abnormalities in gait [5]. Vinca alkaloids primarily impact sensory nerves, resulting in a spectrum of symptoms from mild numbness to intense neuropathic pain. Proteasome inhibitors can cause motor and sensory neuropathy and are frequently used in the treatment of multiple myeloma [6].

So along with medical management, physiotherapy is crucial for improving patients' functional outcomes and managing CIPN-related symptoms. Exercises using the closed kinetic chain (CKC) have shown promising effects on lowering neuropathic pain and improving functional abilities in people with CIPN. The proximal component of the limb moves throughout the exercise, while the distal part remains stationary [7]. CKC exercises improve overall functional mobility and lower the risk of falls by strengthening neuromuscular control, improving proprioception, and stabilising joints. These exercises assist in maintaining independence in daily activities while also strengthening and enhancing muscle coordination [8]. Exercises such as squats, lunges, step-ups, and calf lifts, which concentrate on strengthening the lower limbs, may be beneficial for patients with CIPN [9].

## Case presentation

In order to treat the symptoms of CIPN, a 52-year-old female patient with Stage II ovarian cancer was recommended for neurophysiotherapy after being admitted to the chemotherapy ward for a second cycle of chemotherapy. The patient received a second round of chemotherapy consisting of 150 mg of carboplatin and 50 mg of docetaxel. The patient complained of pain, lower limb weakness, tingling in both feet, and trouble keeping her balance when walking after three days of the second round of chemotherapy. The patient did not have a medical history of infectious diseases, nutritional deficiencies, or diabetes mellitus. So physiotherapy sessions were started.

Clinical findings

A detailed sensory and motor examination was performed. Sensations were diminished. The deep tendon reflexes were diminished. There was generalized weakness in both lower limbs with the strength of major muscles of the lower limb as Grade 3 on Medical Research Council Grading. The modified total neuropathy score (TNS) was 15, i.e. moderate neuropathy. Semmes Weinstein Monofilament Grading was graded 3, suggesting loss of protective sensation. The patient had the highest independent attainable position standing. While walking, she needed mild assistance. On the Berg Balance Scale, the score was 27, which is predictive of the risk of falls. According to the National Cancer Institute - Common Toxicity Criteria (NCI - CTx), peripheral neuropathy was Grade 3, suggestive of severe symptoms limiting self-care; peripheral sensory neuropathy was Grade 2, suggesting moderate symptoms limiting instrumental activities of daily living; and neuralgia was Grade 1, suggesting mild pain. Table 1 depicts manual muscle testing examination findings.

**Table 1:** Manual muscle testing for lower limbs Grade 3: Full range of motion against gravity; Grade 4: Full range of motion against gravity with mild to moderate resistance; Grade 5: Full range of motion against gravity with maximal resistance

Muscles	Right	Left
Hip flexors	3/5	3/5
Hip extensors	4/5	4/5
Hip abductors	3/5	4/5
Hip adductors	3/5	4/5
Knee flexors	4/5	3/5
Knee extensors	3/5	3/5
Ankle dorsiflexors	4/5	4/5
Ankle plantar flexors	3/5	3/5

Intervention

In March 2024, the patient was admitted to the chemotherapy ward for the second cycle of chemotherapy, so to manage CIPN, a referral was made to the Neurophysiotherapy Department. Comprehensive rehabilitation was given for four weeks. Table 2 depicts the physiotherapy rehabilitation protocol.

**Table 2:** Brief summary of physiotherapy rehabilitation TENS: Transcutaneous electrical nerve stimulation; CKC: Closed kinetic chain

Sr No.	Goal	Intervention	Rationale	Dosage
1	To remind the patient of their present state and the potential results of their rehabilitation	Educating patients about the importance of physical therapy and rehabilitation in the treatment of chemotherapy-induced peripheral neuropathy	Help to increase compliance with physiotherapy sessions	N/A
2	To alleviate lower limb pain and tingling sensation	High TENS (50-100 Hz) applied in 4 poles linear pattern over the posterior aspect of the thigh and calf	TENS reduces pain through the pain gate mechanism.	15 minutes once a day
3	To enhance the stability of proximal muscles	Rhythmic stabilization technique for hip flexors and extensors	Balance is improved when agonist and antagonistic activity co-occur (co-contraction).	10 reps with 3 sets
4	To improve the strength of lower limb muscles	CKC exercises: Terminal knee extension exercise, lateral step-ups, wall squats, side stepping, standing heel raise - single leg	Exercises using the CKC have demonstrated enhanced Ia muscle spindle afferent firing. It also enhances the threshold to motion sense, which in turn improves postural control.	10 reps with 2 sets
5	To gain independence in walking	Utilising the contract-relax technique, the D2 flexion-extension pattern was executed.	Isotonic stretching causes muscle contraction along with movement, thus improving balance.	10 reps with 3 sets

**Figure 1:**
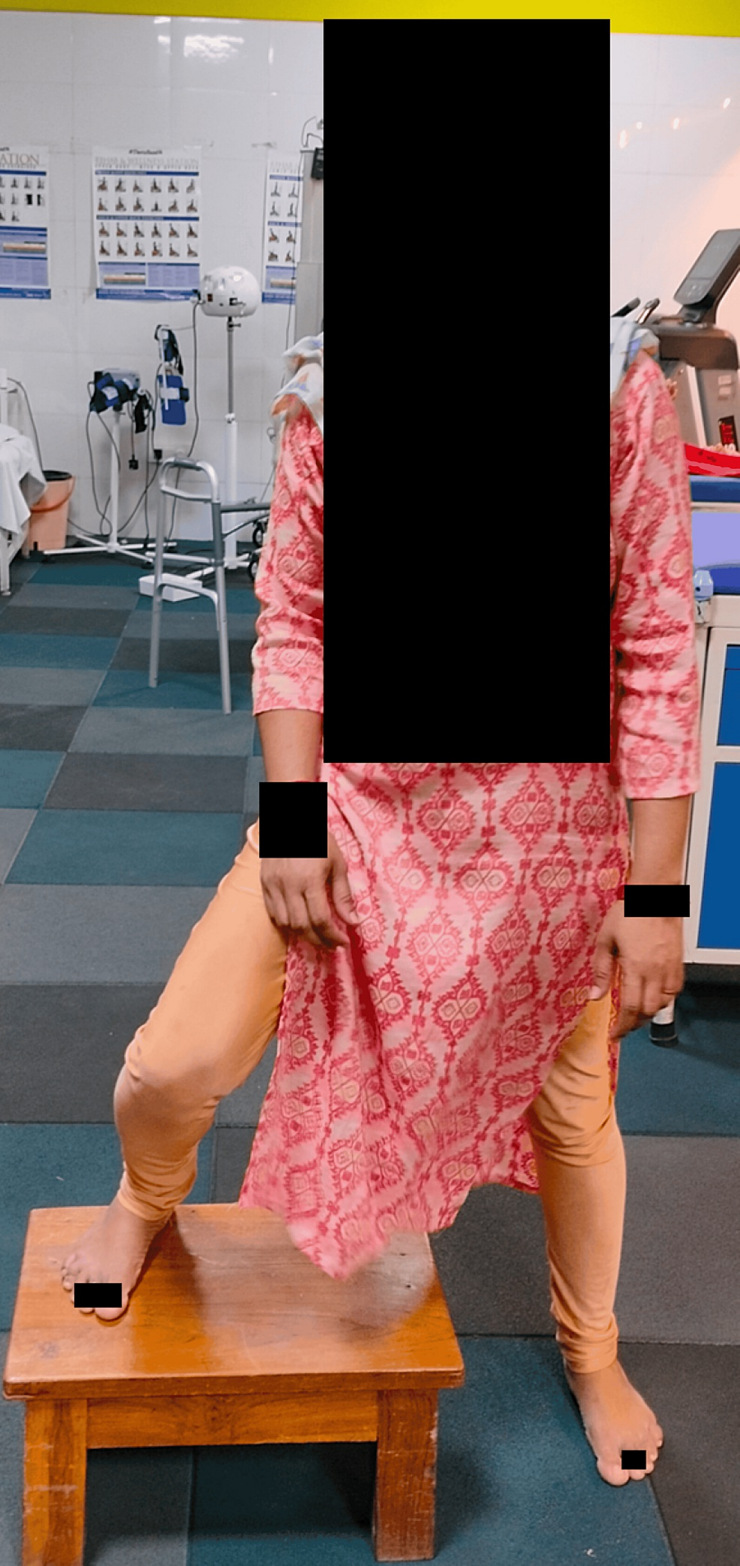
Patient performing the step-up exercise

Figure 1 shows the patient performing the step-up exercise.

**Figure 2:**
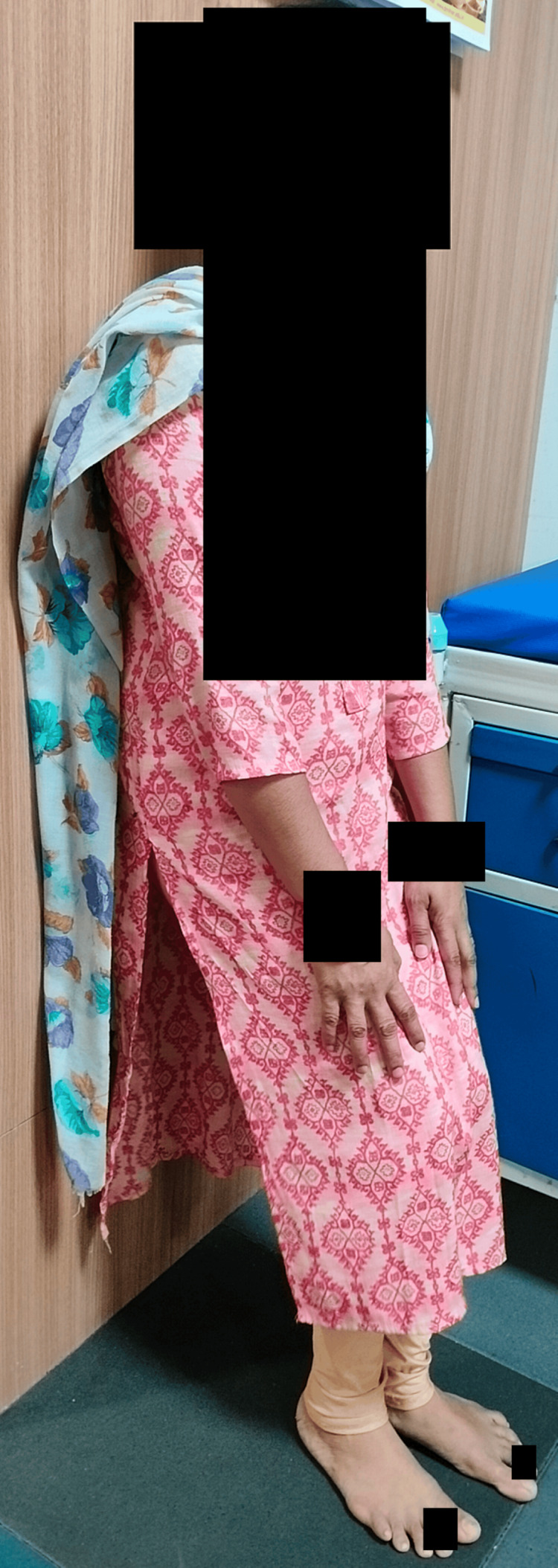
Patient performing wall squats

Figure 2 depicts the patient performing wall-supported squats.

Outcome measures

Pre- and post-physiotherapy rehabilitation, the following outcomes mentioned in Table 3 were taken, which suggested a significant reduction in pain, improvement in neuropathy symptoms, independence while walking, and overall improvement in quality of life. Table 3 shows examination findings for outcome measures.

**Table 3:** Pre- and post-treatment outcome measures FACT/GOG - NTX: Functional assessment of cancer therapy/gynecologic oncology group - neurotoxicity

Sr No.	Outcome measures	Pre-treatment	Post-treatment
1	Modified total neuropathy score	15/24	7/24
2	Visual analogue scale	8	5
3	Semmes Weinstein monofilament test	Grade 3	Grade 5
4	Berg balance scale score	27/56	42/56
5	FACT/GOG - NTX	98/156	114/156

## Discussion

CIPN is a common side effect of chemotherapy that can be quite debilitating for cancer patients. This case study shows how effectively PT interventions can enhance the quality of life, neuropathy score, and balance of a patient with CIPN. It also offers evidence to back up these previous research conclusions [10]. Previous studies have demonstrated the positive impact of physiotherapy therapies on improving patients' quality of life with CIPN. A multimodal exercise plan that includes strength, endurance, and balancing exercises was shown in Kneis et al.'s investigation to significantly improve pain, physical function, and overall quality of life in persons with CIPN [11]. In a similar way, a thorough research carried out in 2014 by Streckmann et al. discovered that exercise therapy, including aerobic, resistance, and balance training, improved the quality of life and reduced the neuropathic symptoms of cancer patients. These outcomes support the findings in our case study about the value of physiotherapy in improving CIPN patients' quality of life [12].

It has been shown that balance training reduces the risk of falls and helps patients with CIPN become more balanced. After conducting a systematic review, Mols et al. discovered that cancer survivors with CIPN had significantly better functional mobility and balance while using balance training therapies such as proprioception and postural control exercises [13]. Furthermore, a meta-analysis by Stout et al. indicated that exercise interventions, such as balance training, decreased the chance of falls and improved balance confidence in cancer patients with peripheral neuropathy. These outcomes support the findings of our case study about the value of balance training in improving balance and reducing the risk of falls [14].

## Conclusions

Cancer patients' quality of life and ability to perform are negatively impacted by CIPN, a common side effect of chemotherapy treatment. Physiotherapy, especially targeted CKC exercises, is essential for controlling symptoms associated with CIPN, achieving better functional outcomes, and promoting the general well-being of patients. Physiotherapy has been demonstrated to help manage CIPN, particularly when it comes to the application of CKC exercises. Exercises known as CKCs, in which the body moves around a fixed distal part, are especially helpful because they improve proprioception and stabilise joints. CKC workouts enhance balance, coordination, and general mobility by emphasising functional movement patterns and building muscle. This strategy improves the quality of life for cancer survivors in addition to easing the discomfort and functional limitations brought on by CIPN. All things considered, adding CKC exercises to physiotherapy regimens presents a viable way to lessen the incapacitating consequences of CIPN.

## References

[1] Zhang X, Chen WW, Huang WJ (2017). Chemotherapy-induced peripheral neuropathy. Biomed Rep.

[2] Anand U, Dey A, Chandel AKS (2023). Cancer chemotherapy and beyond: current status, drug candidates, associated risks and progress in targeted therapeutics. Genes Dis.

[3] Maihöfner C, Diel I, Tesch H, Quandel T, Baron R (2021). Chemotherapy-induced peripheral neuropathy: current therapies and topical treatment option with high-concentration capsaicin. Support Care Cancer.

[4] Zajączkowska R, Kocot M, Leppert W, Wrzosek A, Mika J, Wordliczek J (2019). Mechanisms of chemotherapy-induced peripheral neuropathy. Int J Mol Sci.

[5] Scripture CD, Figg WD, Sparreboom A (2006). Peripheral neuropathy induced by paclitaxel: recent insights and future perspectives. Curr Neuropharmacol.

[6] Park SB, Cetinkaya FA, Argyriou AA, Höke A, Cavaletti G, Alberti P (2023). Axonal degeneration in chemotherapy-induced peripheral neurotoxicity: clinical and experimental evidence. J Neurol Neurosurg Psychiatry.

[7] Niemand EA, Cochrane ME, Eksteen CA (2020). Physiotherapy management of chemotherapy-induced peripheral neuropathy in Pretoria, South Africa. South Afr J Physiother.

[8] Kwon YJ, Park SJ, Jefferson J, Kim K (2013). The effect of open and closed kinetic chain exercises on dynamic balance ability of normal healthy adults. J Phys Ther Sci.

[9] Brett Whalen L, Zachary WW, Kundur P, Angadi S, Modesitt SC (2022). Beneficial effects of exercise on chemotherapy-induced peripheral neuropathy and sleep disturbance: a review of literature and proposed mechanisms. Gynecol Oncol Rep.

[10] Loprinzi CL, Lacchetti C, Bleeker J (2020). Prevention and management of chemotherapy-induced peripheral neuropathy in survivors of adult cancers: ACSO guideline update. J Clin Oncol.

[11] Kneis S, Wehrle A, Müller J (2019). It's never too late - balance and endurance training improves functional performance, quality of life, and alleviates neuropathic symptoms in cancer survivors suffering from chemotherapy-induced peripheral neuropathy: results of a randomized controlled trial. BMC Cancer.

[12] Streckmann F, Kneis S, Leifert JA (2014). Exercise program improves therapy-related side-effects and quality of life in lymphoma patients undergoing therapy. Ann Oncol Off J Eur Soc Med Oncol.

[13] Mols F, Beijers T, Vreugdenhil G, Franse LV (2014). Chemotherapy-induced peripheral neuropathy and its association with quality of life: a systematic review. Support Care Cancer.

[14] Stout NL, Baima J, Swisher A, Stone KW, Welsh J (2017). A systematic review of exercise systematic reviews in the cancer literature (2005-2017). Conte Iss in Cancer Rehab.

